# Phenotype annotation with the ontology of microbial phenotypes (OMP)

**DOI:** 10.1186/s13326-019-0205-5

**Published:** 2019-07-15

**Authors:** Deborah A. Siegele, Sandra A. LaBonte, Peter I-Fan Wu, Marcus C. Chibucos, Suvarna Nandendla, Michelle G. Giglio, James C. Hu

**Affiliations:** 1 Department of Biology, Texas A&M University, College Station, TX USA; 2 Department of Biochemistry and Biophysics, Texas A&M University and Texas AgriLife Research, College Station, TX USA; 3 Institute for Genome Sciences, University of Maryland School of Medicine, Baltimore, MD USA

**Keywords:** Annotation, Phenotypes, Ontology, Biomedical ontologies, Curation, Microbial phenotypes, Microbial genetics, Microbiology

## Abstract

**Background:**

Microbial genetics has formed a foundation for understanding many aspects of biology. Systematic annotation that supports computational data mining should reveal further insights for microbes, microbiomes, and conserved functions beyond microbes. The Ontology of Microbial Phenotypes (OMP) was created to support such annotation.

**Results:**

We define standards for an OMP-based annotation framework that supports the capture of a variety of phenotypes and provides flexibility for different levels of detail based on a combination of pre- and post-composition using OMP and other Open Biomedical Ontology (OBO) projects. A system for entering and viewing OMP annotations has been added to our online, public, web-based data portal.

**Conclusions:**

The annotation framework described here is ready to support projects to capture phenotypes from the experimental literature for a variety of microbes. Defining the OMP annotation standard should support the development of new software tools for data mining and analysis in comparative phenomics.

## Background

Phenotypes are the result of the interaction of a particular genotype with an environment. An organism’s phenotypes will vary in different environments or life stages. Just as we see the arctic fox’s fur change in color and thickness as summer warmth changes to winter cold [1], we can also observe changes in microbes as their environments change. For example, when faced with nutrient depleted environments some bacteria will change their phenotype from vegetative cells and become spores that can survive adverse environments [2]. Other bacteria switch from swimming to swarming motility in viscous environments or when moving across a surface [3, 4]. Likewise, if a change occurs in the underlying DNA sequence of an organism, creating a new genotype, a change in the phenotype may be observed. Linking particular phenotypic changes to changes in specific genes provides the raw material for understanding the vast variety in biological form and function and is key to genetic dissection of biological processes. Microbial genetics has played a central role in the history of molecular biology. The unity of biology is reflected in how insights based on microbial model systems have informed the understanding of the biology of other clades, including humans.

The Ontology of Microbial Phenotypes (OMP) [5] was created for the systematic annotation of the phenotypes of microbes (e.g. bacteria, archaea, viruses, protists, etc.) in a common framework that supports computational data mining and analysis. The current release of OMP contains 1880 terms describing phenotypes associated with all aspects of microbial life (e.g. morphology, growth, metabolism). Each OMP term consists of a term name (or label), definition, and unique identifier. For example, the term with id OMP:0000041 has the name ‘increased cell size’ and the definition “An altered cell size phenotype where the volume of a cell or cells is increased relative to a designated control”. The association of an OMP term id with a particular gene variant or allele indicates that the genotype in question, when found in a particular environment, leads to the phenotype described by the OMP term.

Previously, Chibucos et al. [5] described the ontology design principles we incorporated into developing OMP. Here, we provide a formal description of OMP annotations, extending the concepts initially proposed in Chibucos et al. [5]. The annotation system to be described here can capture a broad variety of phenotypes from type strains, mutants, and genetic suppressors and enhancers in all kinds of microbial systems. The OMP annotation framework and a wiki-based online interface are being used to collect and display microbial phenotype annotations using OMP terms.

## Results

### The elements of an OMP annotation

**Fig. 1:**
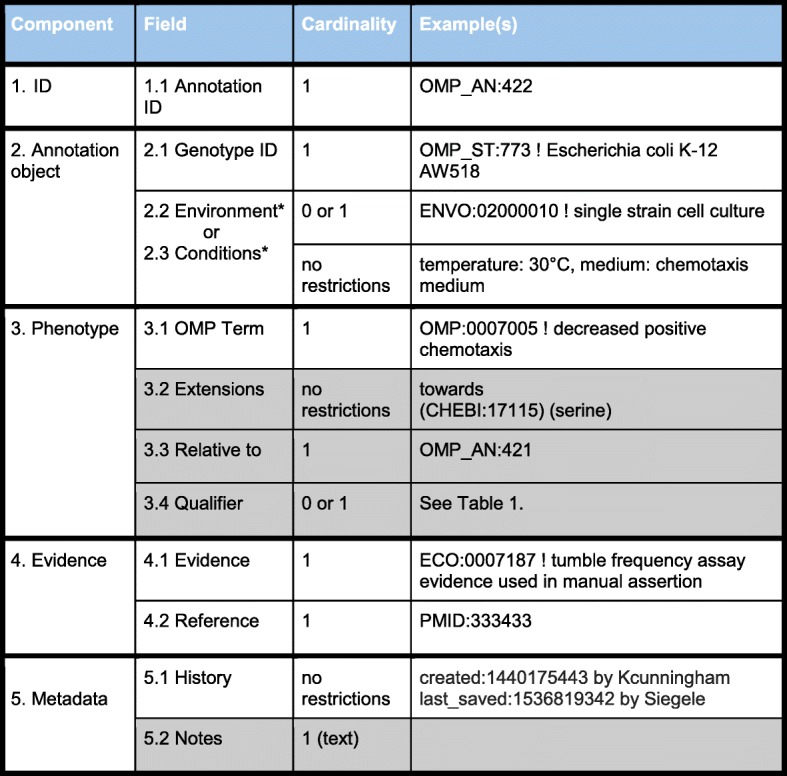
Phenotypes in the OMP wiki. **a** An Annotations table on a Strain page. Two annotations are shown. The interface calculates differences in the genotype and conditions for each annotation compared to the reference annotation. In the second row, the comparison is to a strain that is not isogenic, so multiple allele differences are shown where only one is likely to be causative. **b** Editing interface using TableEdit. This shows how an existing annotation can be edited. OMP and ECO term names are filled in from the IDs by a database lookup. Conditions are entered as multiple key-value pairs where allowed keys are selected from a pull-down menu. These include, ENVO term, temperature, pH, medium, and other. Extensions and Notes are entered as free text

Figure 1 lists the components of an OMP annotation, each of which will be discussed below. Specific fields in Fig. 1 will be referred to in parentheses. We maximize the use of interoperable ontologies and computable identifiers in OMP annotations, however, for some information types we currently use free text content if other more systematic solutions are not yet available.

### Annotation ID

All OMP annotations are assigned a unique ID (1.1), which consists of an OMP_AN prefix followed by an integer and an optional suffix used for annotations made by other groups. These are currently created through the annotation web interface (described below). We assign stable identifiers to each annotation for two reasons: to track corrections to an annotation if needed, and to allow one annotation to make reference to another annotation as described in more detail below.

### Annotation object

Although phenotypes are often discussed in terms of association with genes, in fact phenotypes are manifestations of the combination of genotype, environment, and developmental stage or cell type. In an OMP annotation, genotype and environment information is captured in the Genotype and Environment fields of the phenotype descriptors, while life stage or cell type is captured in the Annotation Extension field.

In this context we need a stable identifier for any genotype that will be subject to phenotype annotation. Ideally, we would reuse an existing resource in the way that GO annotation can reuse identifiers from global resources such as UniProt. A variety of stock centers and collections such as ATCC associate genotypes with a stable identifier. Also, genome accessions at GenBank are available for microbial genomes that have been sequenced. However, these external resources are not sufficient, because a substantial fraction of the literature involves strains that have not been sequenced or have not been deposited in any collections. Because there is no external resource that provides unique identifiers to the wide range genotypes that OMP will be used to annotate, we built this capability into the OMP annotation infrastructure. **Genotype ID** (2.1) is a unique stable ID that has OMP_ST: as a prefix followed by an integer. Each ID is associated with information about a particular microbial substrain, including known alleles, episomes, and ancestry. If available, a source for obtaining the strain is included, along with a reference for where the strain was described in the literature. Where available, stable identifiers from genomes or other resources can be added.

In many high-throughput microbial phenotype studies, where the fitness of a large number of mutants are being compared across a large number of growth conditions, the fitness of each individual mutant is measured relative to the average fitness of all the mutants in the collection rather than to the fitness of the parental strain [6–8]. To capture these relative phenotypes in OMP, we have created special virtual strains that represent the average behavior of the particular collection of mutants used in a particular study. The virtual strain is used as the reference strain that each individual mutant is compared to.

For capture of **environment** (2.2), we prefer to use ontology-based descriptors such as ENVO terms [9, 10]. However, ENVO does not currently contain the terms needed for microbial phenotype annotations. While term development and annotation practice are being worked out with the ENVO team, we use the placeholder **conditions** field (2.3) for a free text description of the environmental conditions where the phenotype was observed.

### Phenotype

Four fields (OMP term, Relative to, Qualifier, and Extensions) combined together form an ontology-based phenotype description.

An **OMP term** (3.1) and **Extensions** (3.2), if any, describe the phenotype. Mungall et al. [11] describe how pre-composition and post-composition of phenotype descriptions are used by different phenotype annotation projects. Briefly, pre-composition consists of using ontology terms with sufficient granularity to capture the desired level of specificity in the annotation system, while in post-composition curators can extend the specificity of annotations by combining less specific terms at annotation time from interoperable ontologies.

The OMP term (3.1) is a pre-composed phenotype description defined by the ontology. Extensions (3.2) is an optional field that can hold zero to many entries to provide more information about the phenotype. Each extension entry is a pairing of a relationship based on the OBO relations ontology [12] and one or more identifiers.

The **Relative to** field (3.3) is used in a specific kind of annotation. There are two kinds of OMP terms to support two kinds of phenotype annotations: independent and dependent [5]. Independent phenotypes are phenotypes of microbes that can be described without reference to another observation. For example, a microbe either has the ability to become motile or is nonmotile. By contrast, description of a dependent phenotype requires reference to another annotation. For example, increased or decreased motility might be observed when comparing a mutant vs wild-type strain or a single strain in different environments. To capture dependent phenotypes, the optional **Relative to** field holds the OMP_AN identifier for the reference phenotype used in the comparison. In many instances, the curator will need to start with creating the annotation for the reference phenotype.

**Qualifier** (3.4, optional) can modify the meaning of the observation. There are currently three allowed values for qualifiers (Table 1).

**Table 1:** Allowed qualifiers and when to use them

Not	Indicates that a phenotype was tested for, but was not observed.
Same phenotype as reference strain	Indicates that a change in genotype does not change the observed phenotype.
Same phenotype as in reference condition	Indicates that a change in environment does not change the observed phenotype.

### Evidence

OMP annotation captures the evidence for a phenotype observation with two fields. **Evidence** (4.1) uses terms from the Evidence and Conclusion Ontology (ECO) [13] to capture the type of experiment used and **Reference** (4.2) provides an identifier for the source of the observation in the literature, usually in the form of a PubMed ID.

### Metadata

**History** (5.1) records revision history of the annotation, including a timestamp for when an annotation was created or changed and who made the changes.

Finally, the annotation system provides an optional free text **notes** (5.2) field for information that could be of value that does not fit into the fields described above. For example, notes could be used to explain revisions or specify where a phenotype is described in a paper. Notes could include links to term requests at OMP, ENVO, ECO, or ChEBI needed to refine the annotation.

### Online system for viewing, creating and editing annotations

**Fig. 2:**
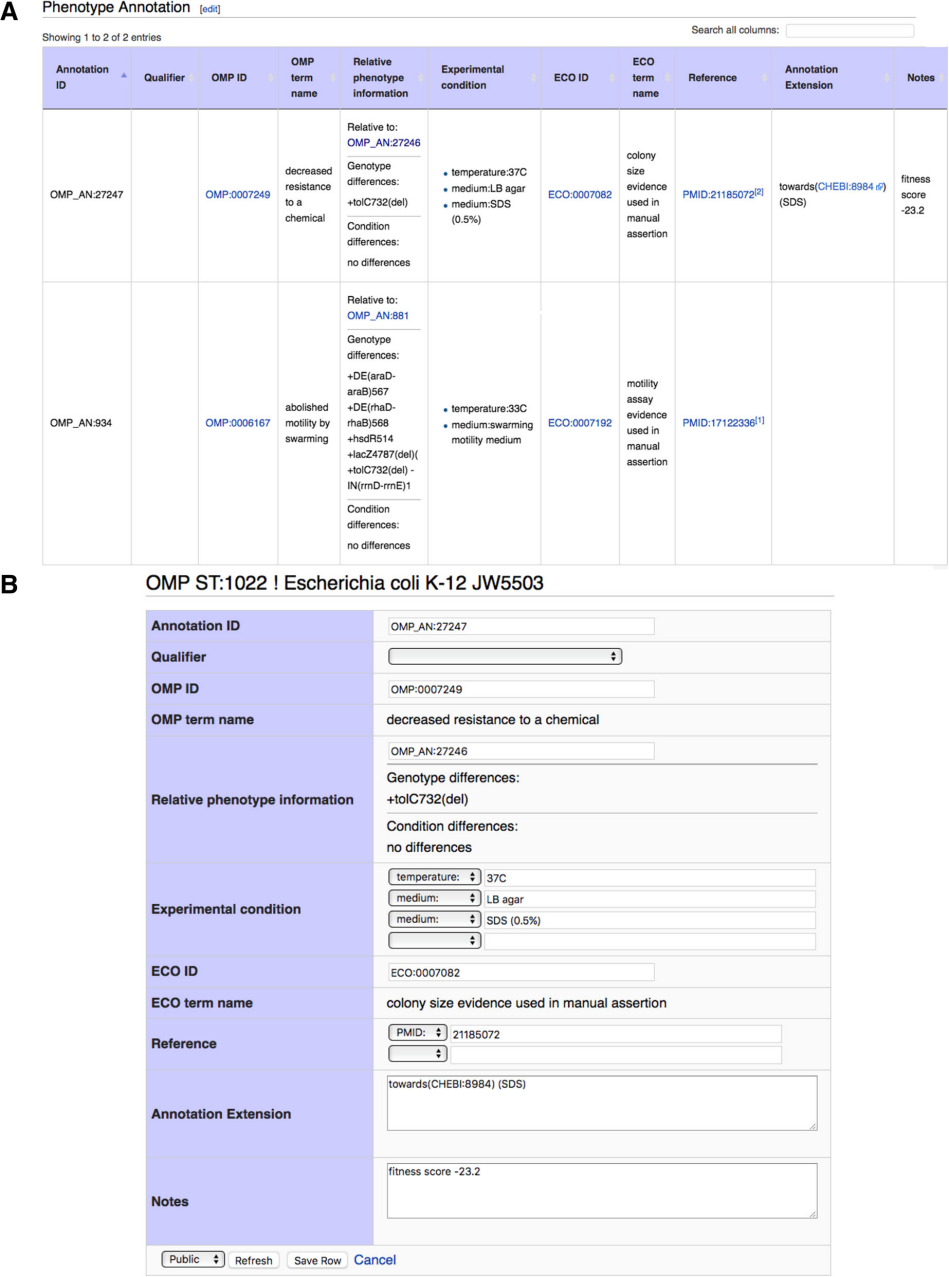
Phenotypes in the OMP wiki. **a** An Annotations table on a Strain page. Two annotations are shown. The interface calculates differences in the genotype and conditions for each annotation compared to the reference annotation. In the second row, the comparison is to a strain that is not isogenic, so multiple allele differences are shown where only one is likely to be causative. **b** Editing interface using TableEdit. This shows how an existing annotation can be edited. OMP and ECO term names are filled in from the IDs by a database lookup. Conditions are entered as multiple key-value pairs where allowed keys are selected from a pull-down menu. These include, ENVO term, temperature, pH, medium, and other. Extensions and Notes are entered as free text.

OMP annotations have been added to the OMP wiki [14], which previously focused on pages for OMP and ECO terms [5]. A system for managing strains and substrains (unpublished) was developed that creates pages for each strain/genotype used in OMP annotation. Strain pages are assigned OMP_ST unique identifiers upon page creation, and the pages include a table for annotations based on the TableEdit Mediawiki extension (unpublished) combined with extra capabilities written specifically for OMP annotation tables. Figure 2 shows an example of an annotation table in the wiki and the editing interface.

Each row in the table represents one annotation, where all the annotations in that table share the OMP_ST ID for the page bearing the table. In addition to the specified annotation component fields described above, the user interface fills in the term name for an entered OMP_ID and an extension to the MediaWiki software calculates differences in the genotype and conditions relative to the reference annotation in the relative_to field as described in Methods. An auto-incremented OMP_AN ID is created when the annotation is saved.

## Discussion

While developing the annotation system for OMP, we examined the annotation formats used by other species-specific microbial phenotype projects. The systems used for *Saccharomyces cerevisiae* [15]*, Schizosaccharomyces pombe* [16], and *Dictyostelium discoideum* [17] appear to be different from one another. The MicrO project [18] provides an alternative ontology for bacterial and archaeal phenotypes and related concepts (e.g. media) but appears to currently emphasize supporting MicroPIE [19] natural language processing, and we did not find a comparable annotation format for use of MicrO. Thus, we decided that developing a distinct universal system to unify annotation would be beneficial.

Insofar as we are building OMP to allow data mining across studies and across microbial species, our annotation system does not capture quantitative fitness scores or measures of growth rates, mutation rates, or other numeric data.

### Pre vs post-composition in OMP

OMP uses a combination of pre- and post-composed approaches to describe phenotypes in annotations. The OMP ontology [5] consists of pre-composed terms that range from broad classification of phenotypes to terms of intermediate specificity where groupings are potentially useful. For example, OMP:0000336 beta-lactam resistance phenotype and its child terms are used when the chemical described in the extension is a beta-lactam, such as penicillin, ampicillin, methicillin etc. Beta-lactam antibiotics are defined by the presence of a beta-lactam ring, which is important for their biological effects on peptidoglycan synthesis in the Bacteria [20]. Phenotypes found for a particular beta-lactam are likely to be informative for the effects of other beta-lactams. Retrieving annotations to these intermediate terms would support analyses that compare and contrast resistance to different members of the antibiotic class, such as the substrate specificity of beta-lactamases [21].

The OMP consortium policy is to limit pre-composition to these intermediate levels, rather than pre-compose a different OMP term for every different beta-lactam antibiotic, even though differences in antibiotic resistance spectrum are potentially useful. Similarly, we do not pre-compose OMP terms for other detailed phenotypes, such as resistance to a particular species of phage, or utilization of a specific nutrient. In these cases, a pre-composed set of terms for every phage and every chemical utilized by a microbe would lead to an astronomical explosion in the size of the ontology.

By contrast, the purpose of the **annotation extension** field in the system described here, which is modeled on the similar extensions used in Gene Ontology Annotation [22–24], is to increase our ability to express specific phenotypes at annotation time without creating new pre-composed OMP terms. Extensions can be used to specify the drug used in an antibiotic resistance phenotype, the cell type where a phenotype is observed (e.g. lethal during spore germination), or other relevant information such as penetrance.

**Fig. 3:**
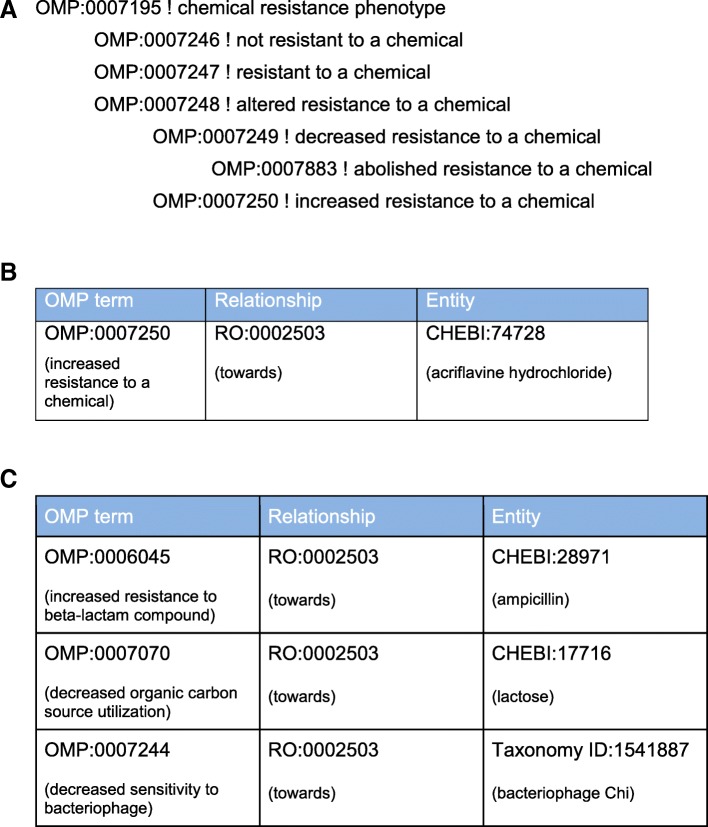
Post-composition with the extensions field. **a** Example of OMP terms with pre-composed groupings at the level of resistance to chemicals. **b** Use of an extension to specify increased resistance to acriflavine hydrochloride using the Relations Ontology term RO:0002503 (towards) to link the ChEBI term CHEBI:74728 (acriflavine hydrochloride) to the OMP term for increased resistance to a chemical. **c** Other uses of the RO:0002503 (towards) relationship include specifying increased resistance to a beta-lactam (ampicillin), decreased organic carbon source utilization (lactose), and decreased sensitivity to a bacteriophage (bacteriophage Chi)

For example, to describe phenotypes relating to resistance or sensitivity to a chemical, OMP contains a variety of pre-composed terms including those shown in Fig. 3a. To identify the specific chemical used in a study, the annotator would add to the annotation extension field a CHEBI ID (or other stable identifier for the chemical), and link the OMP term to the chemical with a “towards” relationship (RO:0002503) from the Relationship Ontology (RO) [12] (Fig. 3b). Figure 3c shows additional examples of how the annotation extension field is used in OMP.

Many microbial phenotypes can be described with pre-composed terms, and some species-specific phenotype annotation systems, such as FYPO [16], are based on extensive pre-composition. The availability of pre-composed terms facilitates community annotation, but can lead to the creation of large numbers of highly specific terms, which can make the ontology unwieldy, especially in an ontology like OMP that coordinates annotation across many taxa.

Post-composition with extensions does not alter the ontology itself. Editing the OMP ontology itself is done as described in Chibucos et al. [5]: term-related requests are gathered via a GitHub issue tracker and changes in the ontology are done using standard ontology editing tools to generate .obo and .owl files, which are periodically released.

### Future directions

#### Populating the corpus of microbial phenotype data

Although we expect that collaborations with other ontology projects and other work in our group will lead to refinements that decrease the use of free text, the annotation system described here should be sufficient to support curation of microbial phenotypes from the literature. Curation projects are ongoing to add phenotypes from high-throughput studies from *E. coli*, *B. subtilis*, *S. pombe*, and *S. cerevisiae*. As each of these presents specific challenges and issues, the details of these contributions to the overall corpus will be described elsewhere.

#### Export formats

A goal for OMP is to provide phenotype data consistent with FAIR principles [25]. Toward the goal of improving interoperability and reuse, we are working on a system for regular releases of the corpus of OMP annotations. We are modeling our first data release specification on the GPAD+GPI system used by GO [22, 26]. For OMP, we would generate a pair of tab-delimited files. One of these would contain the annotation fields specified here, while the second would include information associated with the genotype in the annotation object. The genotype representation system is under development.

As an alternative to tab-delimited text, it should be possible to export OMP annotations and the associated genotype information as JSON or JSON-LD [27].

## Conclusions

We describe a framework for the use of OMP to make phenotype annotations. This system is in active use for the annotation of phenotypes for *Escherichia coli, Bacillus subtilis, Saccharomyces cerevisiae, Schizosaccharomyces pombe,*and other microbes.. A wiki-based online interface allows viewing of annotations and community/collaborative curation of phenotype annotations.

The OMP annotation standard, as defined here, will support the development of new software tools for data mining and analysis in comparative phenomics.

## Methods

The annotation system as described here is a platform-independent specification.

The OMP wiki [14] implementation of the annotation system is based on the open source Mediawiki software platform [28]. The OMP wiki is currently running on Mediawiki 1.31 using php7.2 and MySQL 5.7 with customized extensions to support biological wikis and ontology projects [29] and additional software extensions developed specifically to support OMP projects. The OMP wiki is currently a virtual host on a single Linux server at Texas A&M shared with other projects. Extension code is open source and available at our GitHub repository [30].

The OMP and ECO ontologies are downloaded from our central repositories daily and parsed into a local mysql database, obo_archive, with a custom schema that incorporates version history for every ontology term.

The annotation system within the wiki is controlled by a custom extension for the OMP project, which in turn builds on TableEdit [31], an extension for managing structured tabular data in MediaWiki, and TableEdit-based code modules developed for ontology wiki projects [29]. The template for the annotation form is defined by a page in the wiki, Template:OMP_annotation_table, which controls formatting and callbacks for the displays in Fig. 2a (viewing mode) and b (editing mode). The annotation editing form (Fig. 2b) uses obo_archive to look up current term names when a curator enters OMP or ECO ids.

Each phenotype annotation is stored as a TableEdit row associated with a specific TableEdit table on a genotype page. Each genotype page also contains a TableEdit table with genotype information defined by a different TableEdit template: Template: Strain_info_table. To calculate possibly relevant differences in genotype and conditions, the extension uses the unique annotation id in the Relative to field to find the content of the conditions field in the reference annotation, and the genotype on the page where the reference annotation is stored. The genotype and conditions fields for the reference and dependent annotation are then tokenized with a regular expression and the differences are calculated by comparing arrays of unique tokens for each field.

## Data Availability

Data sharing is not applicable to this article as no primary datasets were generated or analyzed during the current study. Individual annotations can be viewed at the OMP wiki [14]. Plans for dissemination of the annotation sets generated using the annotation system described here are discussed in the text. Software developed for the work described in this article (Mediawiki extensions) are available from our GitHub repository [14].

## Abbreviations

ChEBI: Chemical Entities of Biological Interest
ECO: Evidence and Conclusions Ontology
ENVO: Environment Ontology
FYPO: Fission Yeast Phenotype Ontology
GO: Gene Ontology
OBO: Open Biomedical Ontologies
OMP: Ontology of Microbial Phenotypes
RO: Relations Ontology
